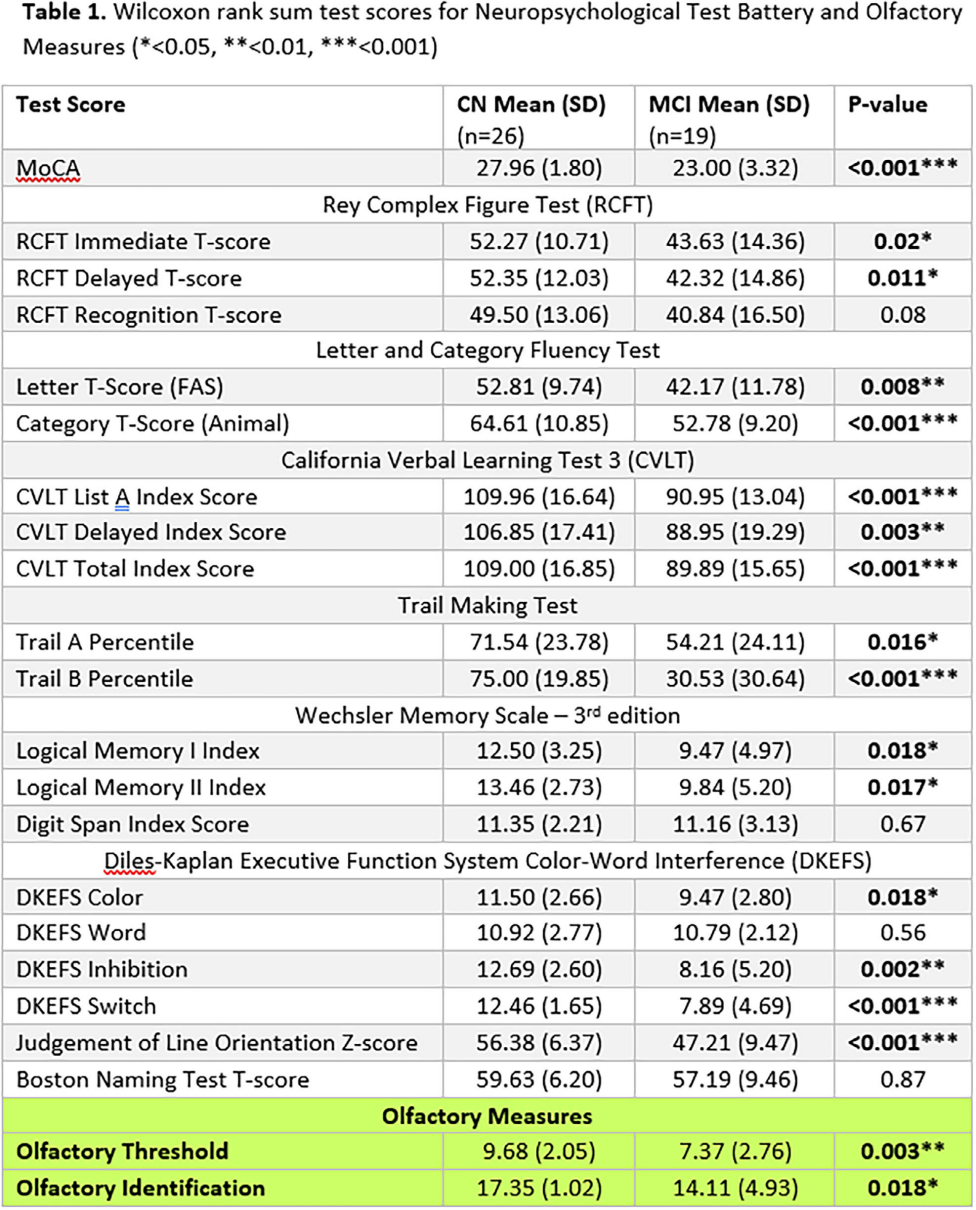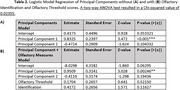# Enhancing Neuropsychological MCI Classification through Olfactory Testing

**DOI:** 10.1002/alz.093428

**Published:** 2025-01-09

**Authors:** Prasanna Karunanayaka, Biyar Ahmed, Rommy Elyan, Senal Peiris, Ran Pang, Sangam Kanekar, Paul Eslinger, Qing Yang

**Affiliations:** ^1^ Pennsylvania State University College of Medicine, Hershey, PA USA; ^2^ Pennsylvania State University College of medicine, Hershey, PA USA

## Abstract

**Background:**

As new therapeutic options emerge, earlier diagnosis is crucial for improving prevention and early intervention. The current standard for screening for MCI and AD involves comprehensive neuropsychological tests prior to performing invasive testing such as lumbar punctures or PET scans. Neuropsychological tests, however, can be variable and confounded by compensatory effects in MCI. Therefore, a more objective means of assessing cognitive deficits would be beneficial. Prominent olfactory deficits have been shown to be prevalent in early MCI and AD and can precede symptoms of memory and cognitive decline. In this study, the relationship between cognition and olfaction was investigated using comprehensive neuropsychological and olfactory testing in cognitively normal (CN) and MCI subjects.

**Method:**

26 CN (18 females, age 65.15 ± 5.44) and 19 MCI (10 females, age 70.10 ± 7.48) participated in this study. Subjects underwent computerized multiple‐choice olfactory identification testing and forced choice olfactory threshold testing. Normalization tables were used to minimize age as a potential confounding variable. To establish a potential composite score, Principal component analysis (PCA) was performed on neuropsychological test scores which was followed by a logistic regression analysis correlating participant status (CN or MCI) versus the PCs with and without olfactory measures. An ANOVA was performed to assess the significance between these two models.

**Result:**

Our findings show significant differences in a wide array of neuropsychological and olfactory measures (Table 1). Measures with a p‐value greater than 0.1 were excluded from the two PCs generated for the PCA. A logistic regression was performed with results and ANOVA findings shown in Table 2.

**Conclusion:**

Olfactory testing improves the ability to differentiate between CN and MCI (Table 2), suggesting that observed olfactory deficits go above and beyond the cognitive impairments measured by neuropsychological tests in the MCI cohort. Our analysis suggests that olfactory testing can improve the sensitivity of the overall assessment of screening for early MCI diagnosis and can be potentially incorporated in a composite score. Since this is an ongoing longitudinal study, testing will be repeated, enabling further insight into the relationship between olfaction, neurodegeneration, and cognition.